# Comprehensive single-cell pan-cancer atlas unveils IFI30+ macrophages as key modulators of intra-tumoral immune dynamics

**DOI:** 10.3389/fimmu.2025.1523854

**Published:** 2025-01-24

**Authors:** Lihe Jiang, Peili Wang, Yixuan Hou, Jingying Chen, Hua Li

**Affiliations:** ^1^ School of Basic Medical Sciences, Youjiang Medical University for Nationalities, Baise, Guangxi, China; ^2^ Medical College, Guangxi University, Nanning, Guangxi, China; ^3^ Key Laboratory of Pollution Exposure and Health Intervention of Zhejiang Province, Interdisciplinary Research Academy, Zhejiang Shuren University, Hangzhou, China; ^4^ Department of General Surgery, Affiliated Hospital of Youjiang Medical University for Nationalities, Baise, Guangxi, China; ^5^ Key Laboratory of Tumor Molecular Pathology of Baise, Affiliated Hospital of Youjiang Medical University for Nationalities, Baise, Guangxi, China

**Keywords:** IFI30, scRNA-seq, spatial transcriptomics, pan-cancer, macrophage, immunotherapy

## Abstract

**Background:**

The convergence of macrophage-targeted strategies with immune checkpoint blockade therapies defines a pivotal avenue in contemporary tumor therapy. Identifying robust genetic regulators in this context is imperative.

**Methods:**

This study elucidates IFI30's role in enhancing Major Histocompatibility Complex II (MHC-II) restriction antigen processing. Despite its recognition in cancer immunotherapy, IFI30 remains a nascent focus. Our approach involves a multi-omics analysis of IFI30 tumor immunological profile in the macrophage-mediated Tumor Microenvironment (TME), spanning various cancers and bolstered by rigorous co-culture laboratory work.

**Results:**

IFI30 predominantly localizes in monocyte/macrophage populations, correlating strongly with immune cell infiltration. Substantiated by single-cell analysis, IFI30 exhibits significant functional enrichment in immune-related pathways. Co-expression with immune-related genes, including MHC elements and immune checkpoints, further validates its relevance.

**Conclusion:**

Our study positions IFI30 as a promising immunotherapeutic target. Pan-cancer analyses and glioblastoma multiforme (GBM) investigations collectively underscore IFI30's potential as a TME modulator, particularly in its interaction with M2-macrophages. IFI30 emerges as a prospective intervention point in the immunotherapeutic landscape.

## 1 Introduction

Despite a plethora of high-precision diagnostic modalities and the emergence of combined remedies for cancer in the last two decades, giving new insights into a medical dilemma that once plagued generations in different dimensions, this disease stays the leading cause of death in most countries (1, 2). When the above scenario is intertwined with the decline in well-being due to distant metastasis of advanced cancers or resistance to specific therapies, the call for more up to date and more advanced treatments is growing. Of late, immunotherapy or, furthermore, immune checkpoint-based immunotherapy has demonstrated a significant clinical advantage in improving the overall prognosis of cancer patients (3–6). However, this advantage seems to be manifested only in a small proportion of treated patients and attenuated by the concomitant occurrence of multiple immune-related undesirable events or short-lived drug resistance reactions (7). With the identification of the tumor microenvironment, this fog dissipates, namely, the existence of a dynamic and sophisticated pattern of multiple cellular or mesenchymal networks in the cancer ecosystem where oncogenes and tumor suppressor genes interact and co-evolve, justifying the generation of events such as immune resistance or immune escape (2, 8, 9). Marked high tumor-associated macrophages (TAM) levels in tumor tissues or any type of immune cells could be an influential contributor to the overarching therapeutic direction. A deeper understanding of the heterogeneity of the tumor immune microenvironment and an assessment of the molecular immune features and penetration levels therein would facilitate the development of a renewed wave of therapeutics to augment prognosis or, at least, to discern innovative immunotherapeutic targets (10, 11).

Interferon-gamma-inducible protein 30 (IFI30), an entity that is intrinsically tethered to lysosomes, exhibits constitutive expression within antigen-presenting cells (APCs), encompassing dendritic cells and monocytes/macrophages. It encodes a pivotal enzyme, namely lysosomal thiol reductase (GILT), which assumes a crucial role in antigen processing and presentation. This enzyme facilitates protein degradation by effectuating the reduction of protein disulfide bonds during endocytosis within a low pH milieu, as corroborated by prior investigations (12, 13). Notably, GILT not only stands as the singularly identified enzyme catalyzing disulfide bond reduction in these processes but also emerges as a linchpin in the processing pathway of regulatory cross-presentation of major histocompatibility complex (MHC) class I and the adaptive immune response to MHC class II restricted antigens, an induction spurred by inflammatory cytokines such as IFN-γ and IL-1 (12, 14). Furthermore, as antecedently documented, IFI30 wields a multifaceted influence, modulating cellular redox homeostasis and thereby governing autophagy, cell activation, and proliferation dynamics. Additionally, it intervenes in T cell tolerance regulation, potentially precipitating autoimmunity (15). For instance, in the context of melanoma, IFI30 has been demonstrated to potentiate the processing and presentation of tumour antigens, TRP1 and TRP2, consequentially augmenting anti-tumour T cell responses and culminating in enhanced patient survival rates (16–18). Analogous potentialities have been postulated for DLBC, BRCA, COAD, GBM, and other neoplastic entities (14, 18). Nevertheless, these extant studies merely skim the surface, leaving an exigency for an in-depth exploration into the specific mechanisms underpinning its effect on the tumour microenvironment (TME) and the discovery of its efficacy commonalities across diverse tumors.

Although the current research on IFI30 in cancer immunotherapy is gaining momentum, the lack of depth and breadth of existing studies is a shortcoming that limits it from becoming a reliable target for immunotherapy. Intriguingly, with the refinement of large-scale gene expression profiling and the advancement of synthetic tools with various multi-omics algorithms, it is feasible to perform pan-cancer expression analysis of IFI30 and assess its correlation with a range of features of the tumor immune microenvironment to maximize the immune potential of this target (19, 20). On top of that, the knowledge of the level of immune penetration at the pan-cancer level and the interactions between cancer cells and various immune cells might be deeply sublimated in the above process in combination with single-cell sequencing technology (21, 22).

**Figure 1:**
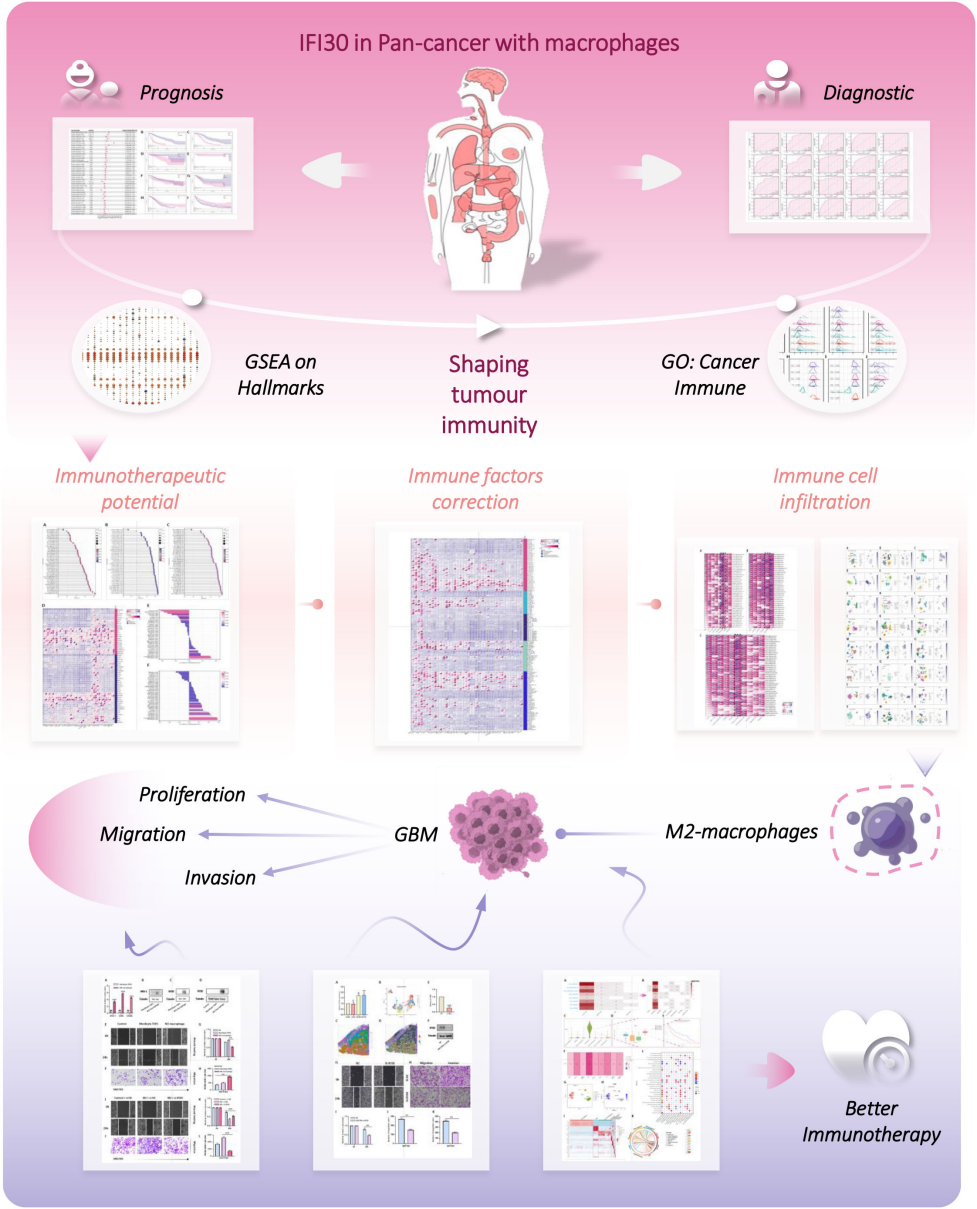
The flow chart of this study.

Consequently, in this study, we comprehensively analyzed the expression of IFI30 in a variety of tumors by calling on available gene transcriptome and single-cell sequencing data resources, and then evaluated its prognostic and diagnostic value. More than this, we initially identified the possible role of IFI30 in tumor immunotherapy by systematic and large-scale pan-cancer GSEA analysis, and validated this conclusion by multiple immune assessment algorithms and correlation analysis of tumor immune micro environment (TIME) features. Furthermore, we further investigated the effect of IFI30 on GBM and modulated this process by entraining macrophages. In conclusion, our study comprehensively depicts the immunotherapeutic properties of IFI30 at the pan-cancer level, a fact that shall provide fresh orientations for immunotherapy. The flow chart of our study is shown in **Figure 1**.

## 2 Materials and methods

### 2.1 Collection and pre-processing of data sets

Pre-collated pan-cancer mRNA seq. data for IFI30 and related clinical data were available through the public database UCSC Xena database (https://Xenabrowser.net/Datapages/) (19, 23). The summary of the pan-cancer dataset is shown in **Supplementary Table S1**. The data were analyzed and visualized through the application of R-studio packages such as “limma”, “ggplot2” and “ggpubr”. Single-cell sequencing data of malignancies mentioned in the research like BCC, BLCA, BRC, CESC, CHOL, CRC, SARC, UCEC, ESCA, GCTB, GBM, HNSC, KIRC, KICH, SKCM, UVM, KIPAN, LIHC, NSCLC, OS, OV, PAAD, STAD, PRAD was obtained from the public database GEO database (https://www.ncbi.nlm.nih.gov/geo/).

### 2.2 The prognosis of IFI30 and the assessment of its diagnostic value

After collating survival data from the various cancer types included in the study and removing samples with missing survival information, pre-treated survival information was obtained. Kaplan-Meier analysis was performed to check the prognostic value of IFI30 for patients with varying disease types, based on standardized hazard ratios (HRs), 95% confidence intervals and p-values, and subsequent analyses were performed using the ‘forestplot’ and ‘survival’ packages. The “pROC” package and the “ggplot2” package were applied to derive the receiver operating characteristic (ROC) of IFI30 in pan-cancer. This study used an AUC value of >0.7 as the reference standard for high diagnostic value.

### 2.3 Systematically functional enrichment analysis in pan-cancer

The “gmt” files of the pre-curated open access set of 50 hallmarks of cancer as well as the immune-related datasets involving GO-BP were obtained from the Molecular Signature Database website (MSigDB, https://www.gseamsigdb.org/gsea/index). The normalized enrichment scores (NES) and false discovery rates (FDR) for each biological process were measured in multiple cancer species under study together with the R packages “clusterProfiler” and “GSVA”, and the R package “ggplot2” was utilized for visualization. Subsequently, according to the magnitude of the Normalized Enrichment Score (NES), the common immune pathways were selected and then presented by plotting with “ggplot2”.

### 2.4 Integrating multiple algorithms into immune features analysis

Stromal, immune, and ESTIMATE scores were generated for each patient with pan-cancer utilizing the R package ESTIMATE based on pre-processed pan-cancer IFI30 gene expression profiles (24). Spearman’s correlation between gene and immune infiltration scores was then calculated for each tumour and the significantly correlated immune infiltration scores were visualized by “ggplot2”. “Timer” “deconvo_epic” and “ MCP -counter” algorithms from the R package “reshape2” and “RColorBreyer” were respectively adopted to evaluate the infiltration scores of different immune cell species in pan-cancer, which were presented visually with the benefit of the R package “reshape2” and “RColorBreyer” (25–27).

### 2.5 Correlational assessment of immune-related factors of tumor

Transcriptome information of IFI30, 24 immune checkpoint pathway Inhibitory genes, 36 immune checkpoint pathway Stimulatory genes, and 150 immune pathway marker genes, that is, 41 chemokine, 18 receptor, 21 MHC, 24 Immuno-inhibitor, and 46 Immuno-stimulator genes, were extracted from the pre-processed pan-cancer expression data (28, 29), with correlation coefficients determined by calculating pearson correlation using the R package “LIMMA”, and the R packages “ggplot2 “and “RColorBreyer” for visualization respectively. The correlation coefficients of the TMB and MSI scores were analyzed from previous studies, then the correlation coefficients between the pre-collated information of transcripts and the MSI and TMB scores of the pan-cancer samples were developed and visualized (30).

### 2.6 Constructing a pan-cancer Sc-seq atlas of IFI30

By applying R package “Seurat” to public databases of BCC, BLCA, BRC, CESC, CHOL, CRC, SARC, UCEC, ESCA, GCTB, GBM, HNSC, KIRC, KICH, SKCM, UVM, KIPAN, LIHC, NSCLC, OS, OV, PAAD, STAD, PRAD. Sc-seq data from tumors were collated and analyzed. “UMAP”, “tSNE”, “SingleR” algorithms were used for dimensionality reduction and cell cluster annotation of the data, respectively. “vlnplot”, “Dimplot” and “Featureplot” were used to visualize the expression characteristics of IFI30.

### 2.7 Processing and visualization of spatial transcriptome data

The spatial transcriptomic data GSE194329 from the primary GBM of the 10xVisium platform was dimensioned by the “SCTransform” function to determine the most variable features and subsequently downscaled by “RunPCA”. The “SpatialFeaturePlot” function was applied to visualize subgroups and genes. Points with less than 500 or more than 6,000 detected genes and points with a mitochondrial count exceeding 20% were filtered out, and cell types were defined at a resolution of 0.8. The “Scanpy” and “stlearn” packages were used for pre-processing, visualization, clustering, temporal analysis and differential expression analysis during data processing. The “stLearn” package was integrated to infer interactions of information on gene expression, spatial position and spatial cell type assignment.

### 2.8 Cultivation of cell lines

The human glioma cells U87MG, LN229, U251MG, SW1783 and THP-1 cells were obtained from the Chinese academy of sciences cell bank, prior to which all cell lines involved in this study were identified by the STR method. Cells were cultivated in a culturing system with 10% fetal bovine serum mixed with RPMI1640, and the overall conditions were maintained in a sterile incubator at a constant temperature of 37°C.

### 2.9 Polarization of macrophage M2 subtypes

THP-1 cells were stimulated with PMA (100 ng/mL) for 24 hours and after being successfully induced with the M0 macrophage phenotype, they were stimulated with IL-4 (20 ng/m L, R&D system) for 48 hours and morphological differentiation to M2 macrophages was observed microscopically and subsequently verified by PCR as well as WB.

### 2.10 Real-time PCR

The manufacturer’s recommended RNAiso-Plus (Takara) was selected for total RNA isolation and extraction, subsequently reverse transcribed into cDNA with the support of a high-volume gene synthesis kit (Takara, China). The optimal conditions for the reaction were determined by referring to the standards prescribed by the reagent vendor, and GAPDH was applied as an internal reference for the abundance assay, with relative expression results calculated with 2^-ΔΔCt method.

### 2.11 Cell transfection

The pre-construction vectors, sh-NC, and sh-IFI30 were constructed and transfection efficiency verified. When cell fusion reached approximately 70-80%, knock-down Cell Transfection Assay was performed with Liposome 3000 Transfection Agent (Invitgen, USA) according to the manufacturer’s instructions.

### 2.12 Western blotting analysis

The well extracted total protein was added to 10% SDS/PAGE gel system according to the recommendation of the reagent vendor, and the protein was transferred to PVDF membrane after being taken to constant 200V ionization. The primary antibody and secondary antibody were incubated individually and followed by washing the PVDF membrane strips and developed color, and the experimental results were recorded after detection of the immunoreactive signal and exposure with the enhanced chemiluminescent kit.

### 2.13 Cell proliferation assay

A pre-treated stable knockdown of IFI30 target cells was inoculated at 3 × 10^3^ cells per well into 96-well culture plates and incubated at 37°C in a germ-free incubator. The absorbance was measured daily at 450 nm using the CCK-8 kit, and the culture was incubated seven times a week, with four secondary wells reserved for each set of experiments.

### 2.14 Transwell, invasion and wound healing assay

The pre-transfected targeted cells were inoculated in a 6-well plate and cultured until the dish bottom was completely covered. The cells were manually scratched to create scratches, and the culture was continued for 48 h with fresh medium. A suspension of 100,000 pretreatment cells (100 μL) and control cells was inoculated into the upper chamber of the tube, and transwell assay and invasion assay were performed with or without matrix gel. After 24 h of growth, the cells were stained with 0.05% crystalline violet dye for 30 mins and photographed for counting.

### 2.15 Statistical analysis

The bioinformatics analysis involved in this study was manipulated by R software with t test and Kruskal-Wallis test applied to assess paired transcriptomic data, and Pearson or Spearman methods were used to evaluate the correlation tests involved, and p values < 0.05 (*p < 0.05) were considered significant, where **p < 0.01, ***p < 0.001, and ****p < 0.0001.

## 3 Results

### 3.1 IFI30 significantly differentially express in pan-cancer

**Figure 2:**
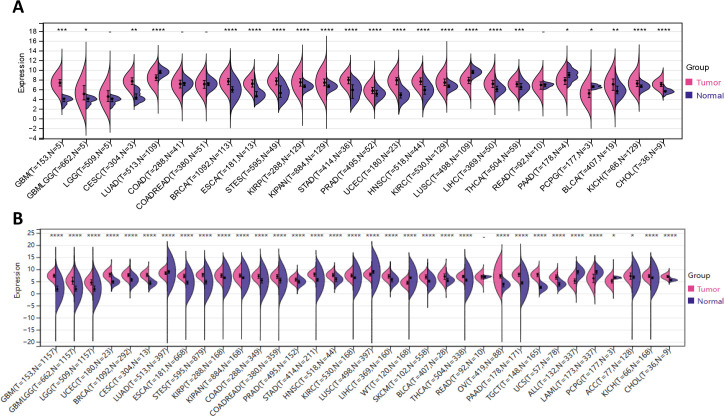
IFI30 is differentially expressed in the common malignant tumors. **(A)** The expression of IFI30 in tumor tissues and paired normal tissues was analyzed using data from the TCGA database. **(B)** Comparison of IFI30 expression differences in TCGA database and GTEx database. *p < 0.05, **p < 0.01, ***p < 0.001, and ****p < 0.0001.

Based on the coupled analyzes of TCGA and GTEx data, we comparatively compared the IFI30 expression in 33 cancers that are currently in common use. Surprisingly, relying solely on the TCGA database, IFI30 was significantly differentially expressed in 22 of the 26 cancers obtained upon removal of sample sizes less than three and thus GBM, GBMLGG, CESC, BRCA, ESCA, STES, KIRP, KIPAN, KIPAN, STAD, PRAD, UCEC HNSC, KIRC, LIHC, THCA, BLCA, KICH, CHOL, etc. (**Figure 2A**). The results were even more impressive when normal samples from the GTEx database were included, and of the 34 cancer types for which expression data were obtained after the above steps, IFI30 was significantly differed in all of these cancers except READ. The top three cancer types were GBM, UCEC, and BRCA (**Figure 2B**). The significant differential expression could be at least somewhat suggestive of an important role of IFI30 in pan-cancer.

### 3.2 Prognostic and diagnostic potential of IFI30

**Figure 3:**
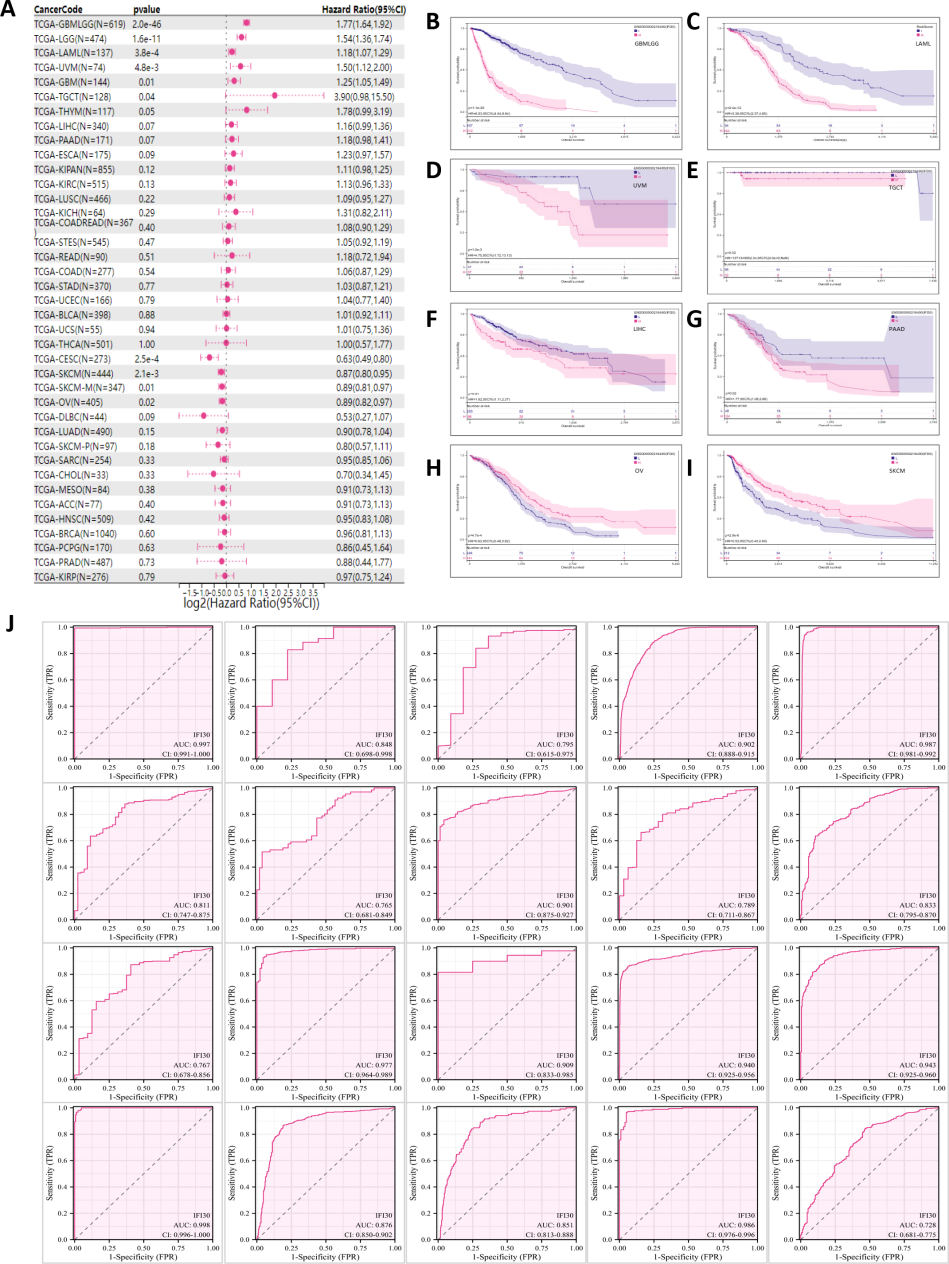
IFI30 could be used as a diagnostic and prognostic marker for a variety of cancer types. **(A)** Correlation between IFI30 expression and OS. **(B–I)** Kaplan Meier curves revealing the relationship between IFI30 expression levels and OS in GBMLGG, LAML, UVM, TGCT, LIHC, PAAD, OV, SKCM. **(J)** Receiver operating characteristic (ROC) curve of IFI30 in pan-cancer. as AUC, area under the curve; CI, confidence interval.

We further examined the significance of IFI30 as a prognostic as well as diagnostic marker in tumour patients by incorporating the TCGA along with the GTEX database and the results were presented in the format of univariate Cox regression analysis and Kaplan-Meier survival analysis. Univariate COX regression analysis demonstrated that in eight tumors, such as GBMLGG, LGG, GBM, TGCT, THYM, UVM, LAML, ALL, high expression of IFI30 was a risk factor for overall survival (OS) in patients with the corresponding tumors, while unlike these results, in the context of tumors such as CESC, SKCM, SKCM-M, OV, and CESC, low expression of IFI30 indicates a worse prognosis (**Figure 3A**). The Kaplan-Meier (Km) survival curves were generally consistent with these results, but we observed that the survival of patients with both LIHC and PAAD decreased with increased IFI30 expression, implying that IFI30 could also act as a risk factor (**Figures 3B–I**). On this basis, we performed a pan-cancer ROC (Receiver Operating Characteristic) profile, which displayed IFI30 as a diagnostic marker in 20 cancers with an AUC > 0. 7: specifically: CESC (AUC = 0.997), CHOL (AUC = 0.848), ESCA (AUC = 0.795), GBMLGG (AUC = 0.902), GBM (AUC=0.987), HNSC (AUC=0.811), KICH (AUC=0.765), KIRC (AUC=0.901), KIRP (AUC=0.789), LIHC (AUC=0.833), OSCC (AUC=0.767), OV (AUC=0.977) PAAD (AUC=0.909), SKCM (AUC=0.940), STAD (AUC=0.943), TGCT (AUC=0.998), THCA (AUC=0.876), THYM (AUC=0.851), UCEC (AUC=0.986), PRAD (AUC=0.728) (**Figure 3J**). The above results largely highlighted the superior diagnostic value of IFI30 for the majority of cancer types.

### 3.3 Systematic functional analysis suggested that IFI30 shapes tumour immunity

**Figure 4:**
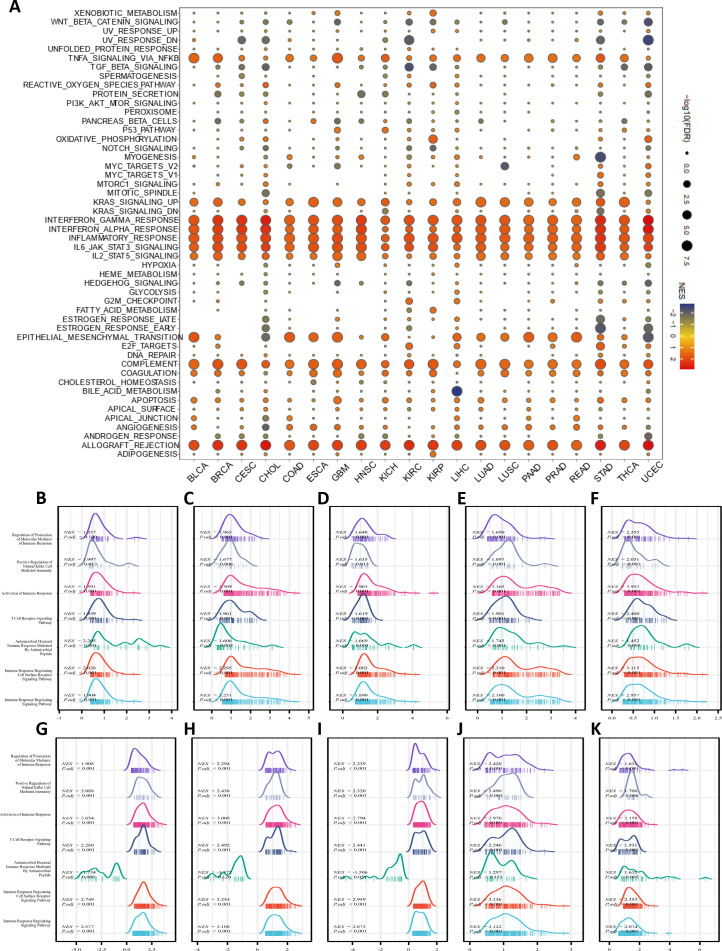
Pathway enrichment analysis of IFI30. **(A)** Hallmarks gene set enrichment analysis (GSEA) of IFI30 in pan-cancer. The size of the circle indicated the FDR value of the enriched term for each cancer, and the color indicated the normalized enrichment score (NES) of each enriched term. **(B–K)** GSEA analysis of immune-related pathways of IFI30 in GBM, LGG, KICH, LIHC, PAAD, SKCM, TGCT, UCEC, UVM, ESCA.

Based on the differential expression of IFI30 in pan-cancer, a GSEA on Hallmarks was performed on 20 cancer types to identify the underlying features of IFI30-associated cancers. Gene Set Enrichment Analysis (GSEA) findings indicated that IFI30 was highly involved in immunomodulatory and inflammation-related pathways in almost every cancer type. The main pathways significantly associated with IFI30 in pan-cancer are “Interferon gamma response, Interferon alpha response”, “inflammatory response, allograft rejection”, and “complement pathway activation” (**Figure 4A**). Moreover, activation of common pathways in some cancers, such as KRAS pathway, activation of IL6-JAK-STAT3/STAT5 pathway and activation of TNFα signaling pathway via NFkB were remarkably correlated. The results of GSEA demonstrated that the expression level of IFI30 showed a significant correlation with the feature sets related to the tumor immune microenvironment and the gene sets associated with ligand-receptor interactions between tumor cells and immune cells, suggesting that IFI30 may potentially be involved in regulating the tumor immune microenvironment and the processes of intercellular interactions. We then further targeted the immunomodulatory pathways within the GO-BP pathway and found that the changes in the expression of IFI30 are correlated with several key indicators and pathways in the tumor immune process. Although it cannot yet be fully confirmed that IFI30 has a significant causal impact, it has provided valuable clues and potential research directions for the subsequent in-depth exploration of the specific mechanism of IFI30’s role in tumor immunity. In cancers with significant prognostic survival relevance, such as GBM, LGG, KICH, LIHC, PAAD, SKCM, TGCT, UCEC, UVM, ESCA, the mentioned pathways were significantly co-regulated (**Figures 4B–K**). For example, “Regulation of Production of Molecular Mediator of Immune Response”, “Positive Regulation of Natural Killer Cell Mediated Immunity”, “Activation of Immune Response, T Cell Receptor Signaling Pathway”, “Antimicrobial Humoral Immune Response Mediated by Antimicrobial Peptide”. These results motivated us to further investigate the involvement of IFI30 in the tumor immune response and microenvironment.

### 3.4 Implications for ICI treatment implied by correlation analysis with the TIME

To further refine our understanding of the immunological role of IFI30 in the neoplastic setting, we calculated Pearson’s correlation coefficient of IFI30 in individual tumors with immune infiltration scores to determine the statistically significant correlation with immune infiltration scores. The StromalScore (**Supplementary Figure S1A**), ImmuneScore (**Supplementary Figure S1B**), and ESTIMATEScore (**Supplementary Figure S1C**) presented significant positive correlations in all 43 tumors involved in the score. More specifically, the most significant correlations with the StromalScore (R > 0.70) were GBMLGG (R = 0.82), LGG (R = 0.77), READ (R = 0.72), GBM (R = 0.71), and PAAD (R = 0.70). Cancers with the most significant correlation with ImmuneScore (R > 0.80) were TGCT (R=0.87), BLCA (R=0.87), SKCM (R=0.86), GBMLGG (R=0.86), LGG (R=0.84), SARC (R=0.83), LUSC (R=0.83), GBM (R= 0.82), UVM (R=0.81), and HNSC (R=0.80). Additionally, GBMLGG (R=0.87), TGCT (R=0.85), BLCA (R=0.84), SKCM (R=0.84), LGG (R=0.82), GBM (R=0.81), READ (R=0.80) were significantly and positively correlated with ESTIMATEScore. That significant correlation of IFI30 with immune infiltration score led us to further explore its relationship with immune detection sites, and the results displayed a significant positive correlation of IFI30 with the 60 immune checkpoint pathway genes included in the analysis overall. Almost the vast majority of this positive correlation was dominated by the immune checkpoint stimulation pathway, the three ICI stimulation genes, ITGB2, CXCL10, and CCL5, were significantly positively correlated with IFI30 in almost all included cancer types, while the two ICI inhibitory genes, HAVCR2, and SLAMF7, exhibited significantly negative correlations with these cancer types, the future exploration of these five genes further exploration might account for novel orientations of ICI drug resistance or sensitization (**Supplementary Figure S1D**). Moreover, we emphasized on CD274, CTLA4, and PDCD1, all of which are currently under intensive study, and demonstrated that the above three ICI genes presented a significant positive correlation with IFI30, except for ALL, LAML, and DLBC, which are three hematological malignancies. The results indicated that IFI30 might be crucial in immunotherapy. To this end, basing on the existing consensus that TMB and MSI could significantly influence ICI therapy, we also investigated the correlation between the above and IFI30 in pan-cancer, showing a significantly positive correlation between TMB and IFI30 in 10 tumors, namely CESC, COAD, BRCA, STES, SARC, STAD, UCEC, THYM, READ, and a significant negative correlation in KICH, GBMLGG, TGCT, CHOL, and OV (**Supplementary Figures S1E, F**). Unlike TMB, IFI30 was only significantly negatively correlated with MSI in GBM, UVM, MESO, ACC, PCPG, TGCT, LIHC tumors, while it was significantly positively correlated with other tumors.

### 3.5 Correlation analysis with immunomodulatory elements

From our speculation, we concluded that the significant correlation of IFI30 with the immune microenvironment might be developed through interactions with immune-related factors. To this end, we further compared the correlation of IFI30 with 150 immune pathway marker genes. These five immune pathways were, chemokine (41), receptor (18), MHC (21), Immunoinhibitor (24), and Immunostimulator (46) (**Supplementary Figure S2**). The results of the correlation analysis implied that the above genes demonstrated significant correlations with IFI30 in most of the tumor types included in the study. It is worth mentioning that the most significant of the five segments was MHC-related genes, and at the pan-cancer dimension, IFI30 showed significant positive correlations with MHC-related genes in different malignancies, except for ALL as well as DLBC. Among them, the most marked cancers were LGG, GBM, TGCT, and the least significant ones were chemokine-related genes, and among them, CCL28, CXCL14, CXCL17, CCL16, CCL27, etc. demonstrated no significant correlation with IFI30 in most cancers. In addition, IFI30 correlated significantly with most immune-related factors in LUAD, PAAD, PRAD, OV, UVM and other malignancies, suggesting that the interaction between IFI30 and these immune factors might be an essential factor influencing the microenvironment of these malignancies, while in THYM, LAML, ALL, DLBC, ACC, KICH, UCEC, CHOL and other tumors, this correlation was not significant.

### 3.6 The properties of IFI30 in the TIME

**Figure 5:**
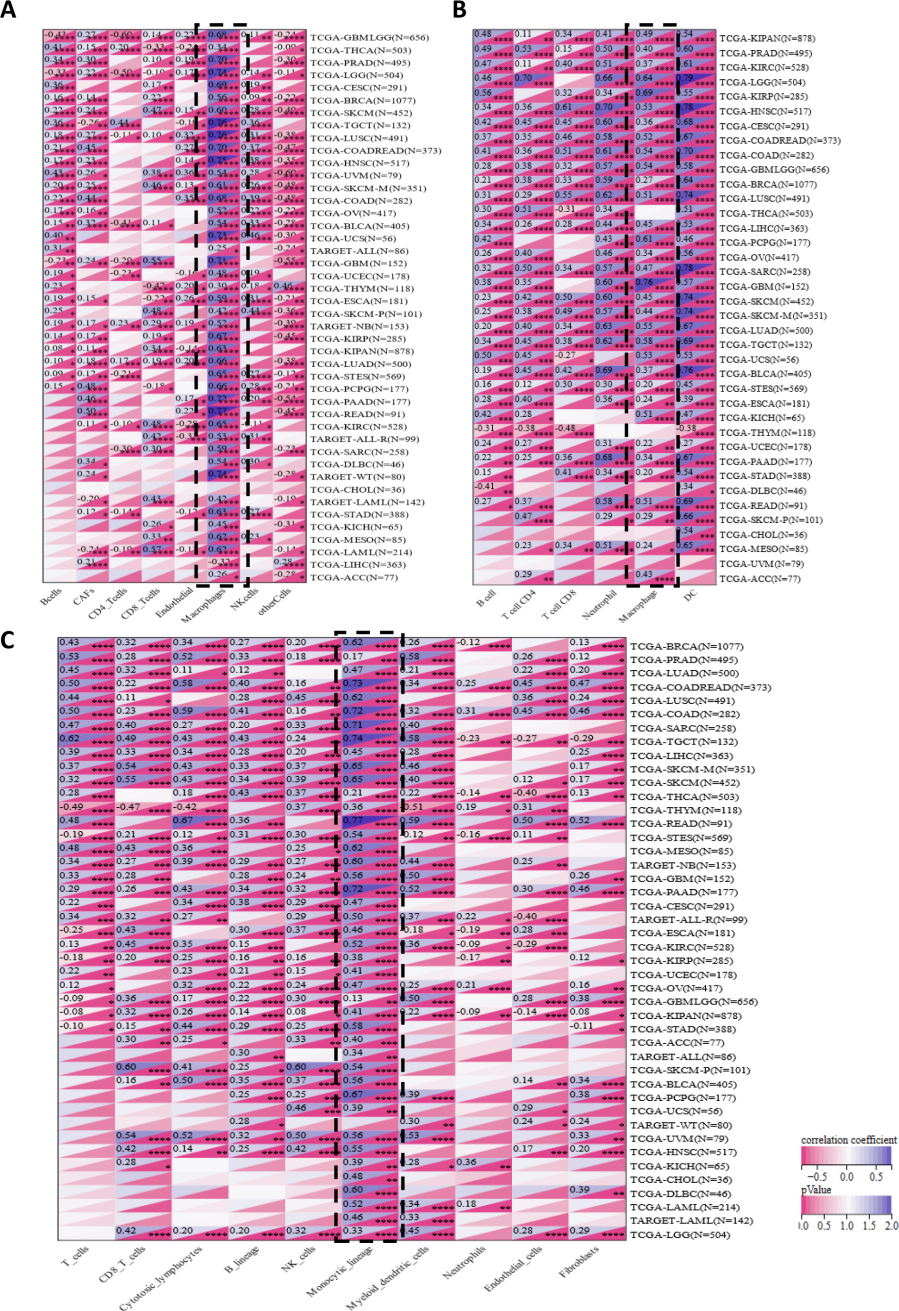
A correlation analysis of IFI30 with the level of immune cell infiltration implying its close association with monocyte/macrophage subpopulations. **(A)** IFI30 was closely related to the immune infiltration level in cancers analyzed via EPIC, **(B)** TIMER and **(C)** MCP-counter algorithms. *p < 0.05, **p < 0.01, ***p < 0.001, and ****p < 0.0001.

In order to further investigate the effect of IFI30 in tumor immunity and to more comprehensively and deeply probe the relevance of IFI30 to immune infiltration, we analyzed the expression of IFI30 at the level of immune cells by utilizing transcriptomic data using three immune algorithms (EPIC, TIMER, MCP-counter) that have been widely available today. As far as the EPIC algorithm is concerned, the infiltration scores of the eight immune cells included in the comparison, that is, B cells, CAFs, CD4_T cells, CD8_T cells, Endothelial, Macrophages, NK cells, and other Cells, were significantly correlated with IFI30 in 43 cancer species. Among them, it was more obvious that macrophages were the dominant group of the eight cell types, presenting a significant positive correlation, and this positive correlation was more significant in PRAD, LGG, TGCT, LUSC, HNSC, PAAD, READ (R > 0.7). In contrast, the negative correlation with CD4_Tcells was more pronounced, but this negative correlation was not quite noticeable at the pan-cancer level (**Figure 5A**). We then performed immune cell infiltration analysis at the transcriptome level following the TIMER algorithm, with differing results compared to the EPIC. Among the infiltration scores of B cell, T cell CD4, T cell CD8, Neutrophil, Macrophage, and DC cell subsets included in the comparison, DC cells were the most significantly correlated module with IFI30. However, in similarity to the significant correlation of DC, Macrophage equally had a significant correlation with IFI30 in pan-cancer. The top three cancer types were, GBM, LGG, and KIRP, which were close to the immune infiltration results implied by EPIC, suggesting an overwhelming presence of IFI30 in the immune microenvironment of these three cancers (**Figure 5B**). Notably, the TIMER algorithm disclosed the possibility that Neutrophil is equally correlated with IFI30, and the MCP-counter algorithm likewise revealed that monocyte macrophage lineage is the most significantly associated cell cluster with IFI30 in the tumor microenvironment, which is consistent with the findings of both algorithms as a whole and corroborates the above results, meaning that IFI30 is highly associated with macrophages in pan-cancer and might potentially exert an influence on the course of the tumor immune microenvironment through its reciprocal relationship with macrophages (**Figure 5C**). Not only that, at the level of individual cancer species, our study revealed that IFI30 did not demonstrate a robust correlation with immune cell infiltration in malignant tumors such as LAML, DLBC, ALL and UVM, and this specificity might afford a varied perspective for further studies on the utility of IFI30 in the immune microenvironment in the coming days.

### 3.7 Pan-cancer Sc-seq atlas reveals that IFI30 is overexpressed on macrophages

**Figure 6:**
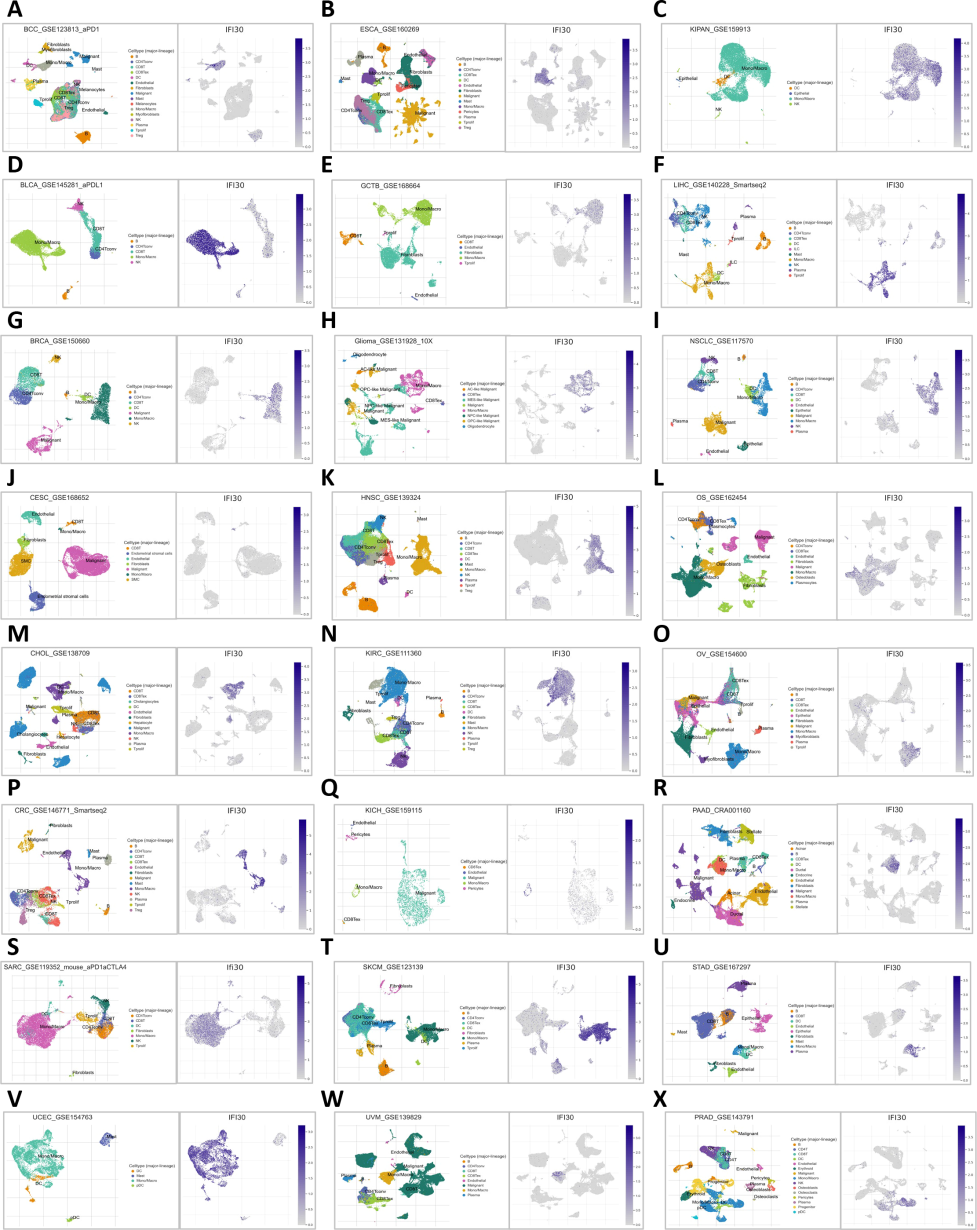
Distribution of IFI30 expression in UMAP of common tumors. **(A)** BCC; **(B)** BLCA; **(C)** BRCA; **(D)** CESC; **(E)** CHOL; **(F)** CRC; **(G)** SARC; **(H)** UCEC; **(I)** ESCA; **(J)** GTCB; **(K)** GBM; **(L)** HNSC; **(M)** KIRC; **(N)** KICH; **(O)** SKCM; **(P)** UVM; **(Q)** KIPAN; **(R)** LIHC; **(S)** NSCLC; **(T)** OS; **(U)** OV; **(V)** PAAD; **(W)** STAD; **(X)** PRAD.

To further understand the underlying contribution of IFI30 in the immune microenvironment, we further constructed a pan-cancer single cell sequencing atlas based on existing public database resources covering BCC, BLCA, BRC, CESC, CHOL, CRC, SARC, UCEC, ESCA, GCTB, GBM, HNSC, KIRC, KICH, SKCM, UVM, KIPAN, LIHC, NSCLC, OS, OV, PAAD, STAD, PRAD and other malignancies (**Figures 6A–X**). As suggested by the transcriptomic data above, the results showed that IFI30 was expressed to varied degrees in a variety of immune cells. Taking the single cell data from BLCA as an example, UMAP showed that IFI30 was presented in five cell clusters including CD4 T cells, NK cells, but we were concerned that the Mono/Macro cell cluster presented a significantly higher expression of IFI30. Similar results were observed in the other 23 tumors included in the study, which to a large extent confirmed the findings of our transcriptomic immune infiltration analysis above that IFI30 is mainly expressed in macrophages and presumably its role in tumour immunity is achieved by influencing the function of macrophages. However, it is worth mentioning that not in all cancer species, macrophage clusters were the dominant cell population, and in PAAD as well as STAD, IFI30 expression was close in macrophages as well as DC cells, suggesting that DC cells might be equally involved in the process of IFI30 influencing the immune microenvironment.

### 3.8 The impact of IFI30 on macrophages is even more accentuated in GBM and significantly correlates with prognosis

**Figure 7:**
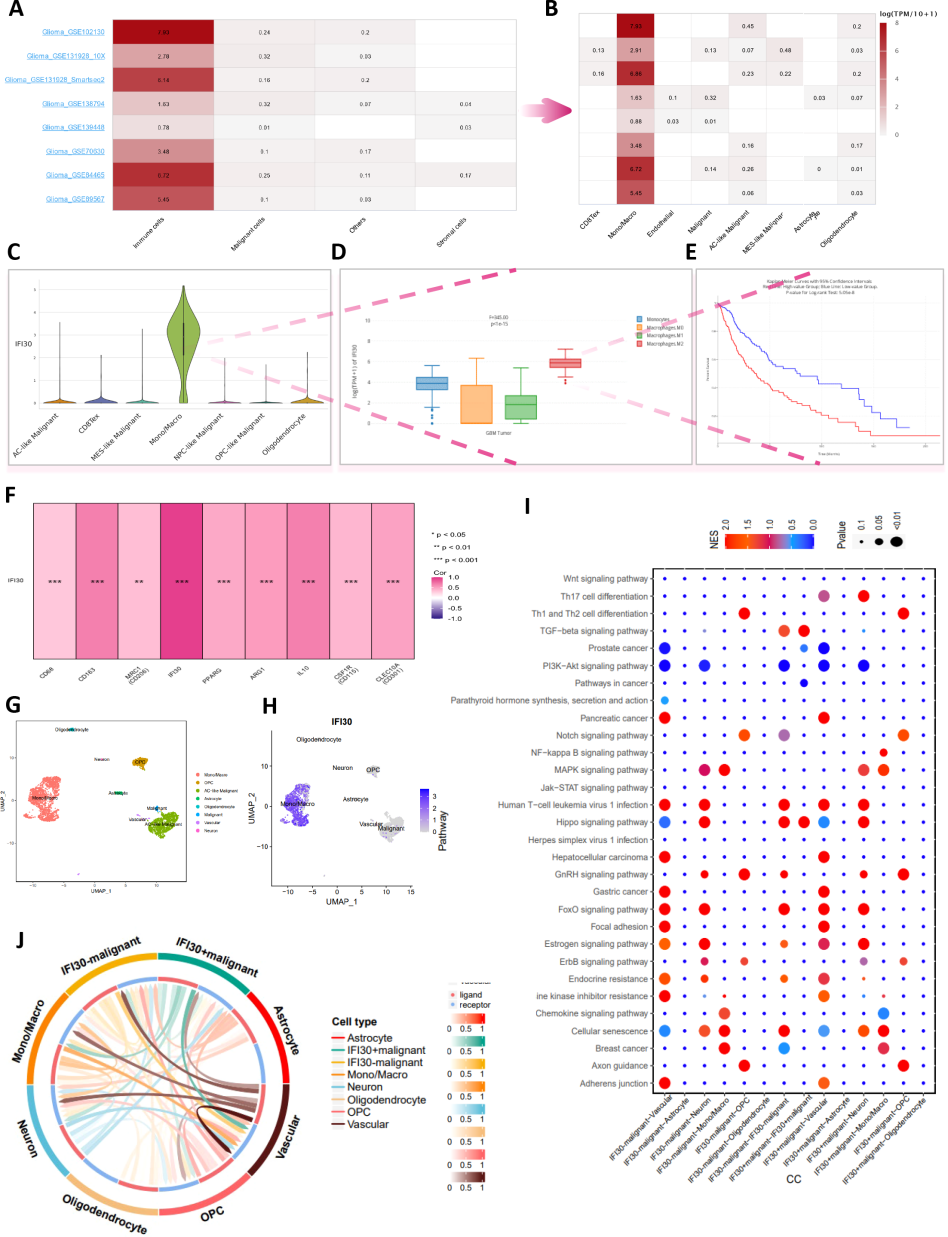
In-depth single-cell data analysis reveals the relation of IFI30 with macrophages in GBM. **(A)** Multiple single-cell datasets reveal that IFI30 is deeply related to immune cells; **(B)** Monocytes/macrophages are the most closely related immune cells to IFI30; **(C)** Violin plots reveal the relative expression of IFI30 in diverse immune cells; **(D)** Box line plots reveal differences in the expression of IFI30 in different subtypes of macrophages; **(E)** IFI30 and M2-macrophages together contribute to the poor prognosis of GBM patients; **(F)** Correlation of IFI30 with M2-macrophage surface marker; **(G, H)** UMAP plots of the single-cell dataset GSE84465; **(I)** Marker genes of signature in different cellular subpopulations; **(J)** Functional enrichment analysis between high- and low-IFI30-expressing subpopulated cells.

Further analysis of available open-access glioma single-cell sequencing data revealed that IFI30 expression was positively correlated with immune cells and, echoing the conclusions drawn above, that macrophage composition made up a large proportion of these immune cells (**Figures 7A, B**). We examined the expression of IFI30 in different cell subpopulations in the GSE102130 single-cell dataset. We found that this difference was still the most significant in macrophages. Subsequently, by utilizing the deconvolution algorithm and introducing the TCGA dataset, and incorporating survival information, we observed that, from the transcriptome perspective, the expression level of IFI30 was the highest in M2 macrophages. Moreover, the synergistic effect of the IFI30 expression level and M2 macrophages indicated a poor prognosis for patients, which implies that IFI30 may alter the immune microenvironment of glioblastoma by influencing M2 macrophages. (**Figures 7C–E**). As a further analysis focused on the correlation of well-known markers for M2 macrophages, recognized M2 macrophage markers including CD68, CD163, MRC1, PPARG, ARG1, IL10, CSF1R, CLEC10A displayed significant correlation with the expression levels of IFI30, which was derived from bulk of TCGA data evidence (**Figure 7F**). To better elaborate this relationship, we then performed a more detailed analysis of GSE84465, a dataset with cellular subgroups shown in (**Figures 7G, H**). We then compared the pathway enrichment analysis of cell subpopulations with high and low IFI30 expression, and the results revealed that Hippo signaling pathway, EGFR tyrosine kinase inhibitor resistance, Cellular senescence, ErbB signaling pathway and other pathways would exhibit differential expression because of IFI30 (**Figure 7I**). Further cell communication analysis suggested that malignant cells with high IFI30 expression tended to exhibit more communication signals with macrophages, and all of the above evidence confirmed the opinion that the effect of IFI30 in GBM on macrophages was more prominent and significantly correlated with prognosis (**Figure 7J**).

### 3.9 IFI30 contributes to the malignant progression of GBM by affecting M2 macrophages

**Figure 8:**
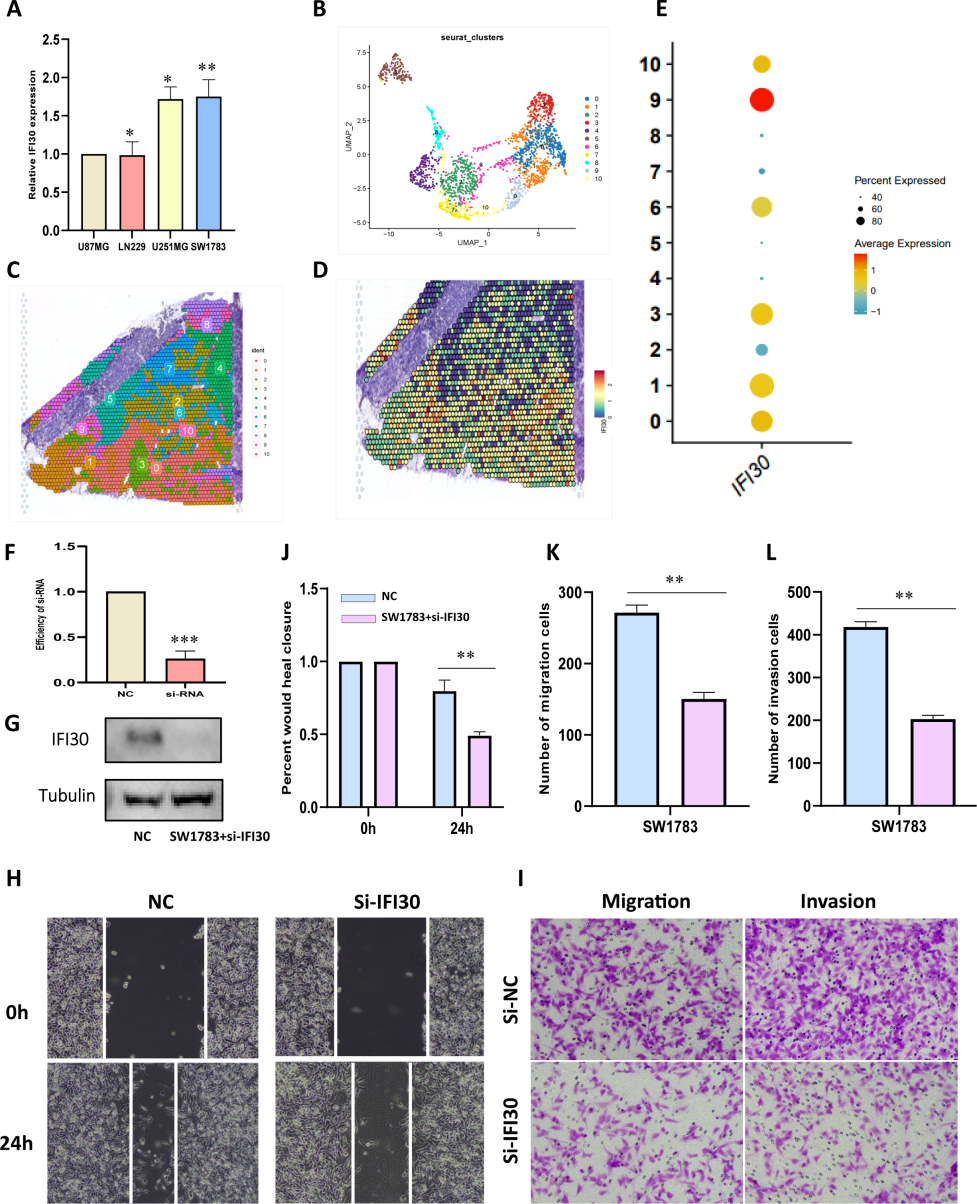
IFI30 impacts the proliferation, migration and invasion of GBM cells. **(A)** IFI30 expression in diverse GBM cells; **(B)** UMAP plot showing the 11 clusters identified by stRNA-seq; **(C)** Spatial plot demonstrating the 11 clusters identified by stRNA-seq; **(D)** Expression level of IFI30 at the spatial transcriptome level; **(E)** Expression of IFI30 in distinct subpopulations of the spatial transcriptome; **(F)** RT-PCR to validate the knockdown efficiency of si-IFI30; **(G)** WB assay to validate the knockdown efficiency of si-IFI30; **(H)** Scratch assay to detect the migratory and invasive abilities of the cells; **(I)** Detection of the migratory and invasive abilities of the cells by the transwell assay; **(J)** Histograms to show the relative mobility of the SW1783 cells; **(K, L)** histograms to show the number of migrated, invaded cells. *p < 0.05, **p < 0.01, and ***p < 0.001.

To further generate laboratory evidence for the above data analysis, we measured the expression levels of IFI30 in four existing GBM cells and displayed the highest expression of IFI30 in SW1783 cells, which were selected for the subsequent mRNA knockdown assay (**Figure 8A**). To better observe the expression level of IFI30 in the GBM tissue environment, we also integrated the spatial transcriptome data GSE194329, and the quality control process of the data is described in (**Supplementary Figure S3**). We observed that IFI30 was differentially expressed in 11 cell subpopulations of the spatial transcriptome data after dimensional reduction clustering, with the most significant expression in subpopulations 0, 1, and 3. According to the original article we could infer that the expression of IFI30 was mainly located in the central tumor location (**Figures 8B–E**). To this end, we further designed siRNA to knocks down the expression level of IFI30, and both RT-qPCR and Western blotting experiments showed that the level of IFI30 was effectively reduced (**Figures 8F, G**). Subsequent scratch as well as migration invasion assays demonstrated that the decrease in IFI30 expression was accompanied by a decrease in GBM cell viability (**Figures 8H–L**).

**Figure 9:**
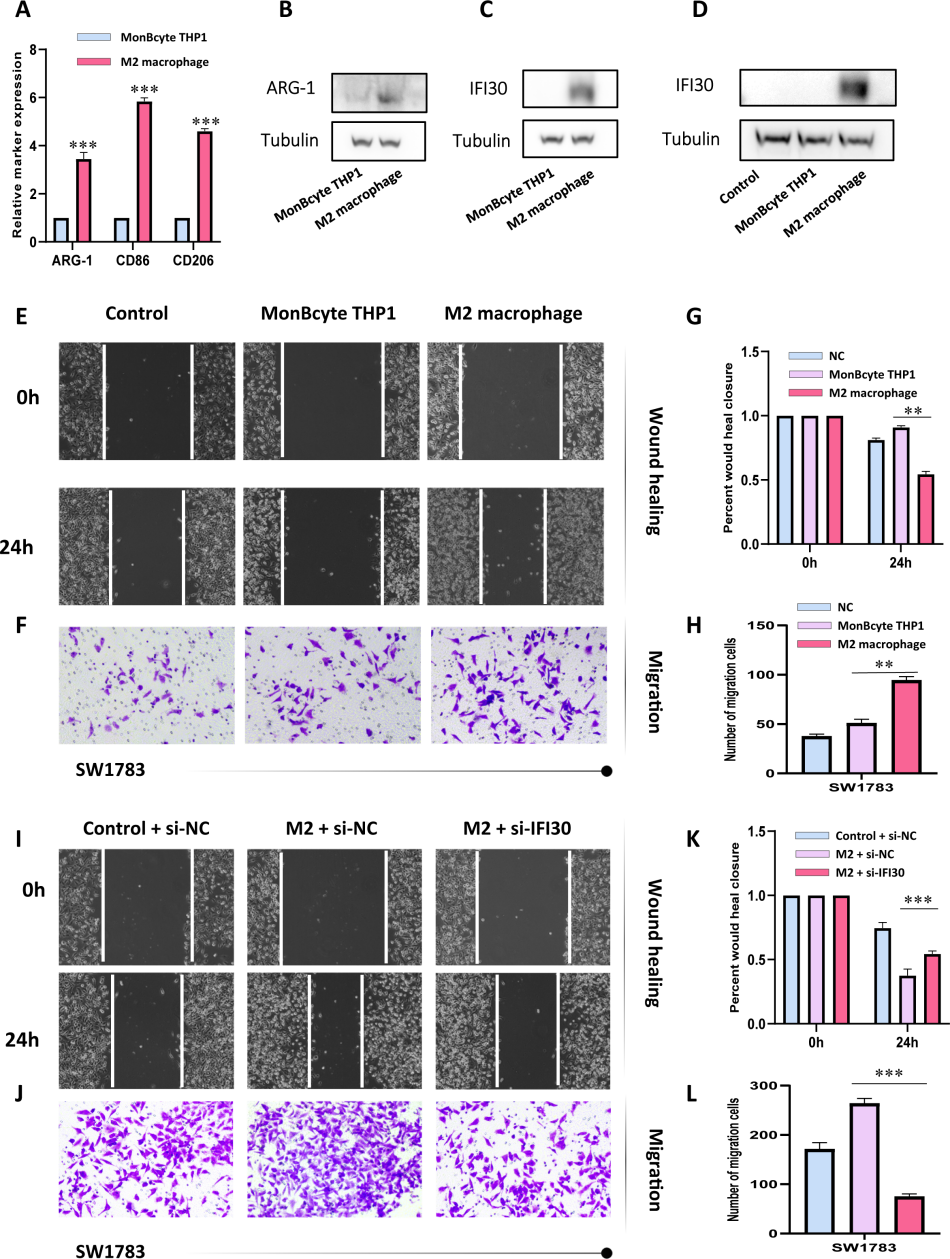
IFI30 is able to influence the growth of GBM cells by interacting with M2 macrophages. **(A)** RT-PCR verified the generation of M2-macrophages; **(B)** WB assay verified the expression level of surface marker-ARG1 in M2-macrophages; **(C)** IFI30 was significantly expressed in M2-macrophages; **(D)** Macrophages co-cultured with SW1783 cells expressed remarkably high levels of IFI30; **(E)** Scratch assay detected the migration and invasion ability of SW1783 cells after co-culture with MonBcyte-THP1 as well as M2-macrophage; **(F)** Migration ability of cells after co-culturing was detected by the Transwell assay; **(G)** Histograms showing the relative mobility of SW1783 cells; **(H)** Histograms showing the number of SW1783 cells migrating under the co-culture system; **(I)** Scratch assays to detect changes in migration and invasion ability after knockdown of IFI30 after co-culturing; **(J)** Cell migration ability after knockdown of IFI30 after co-culturing was detected by Transwell assay; **(K)** Histogram showing the relative mobility of SW1783 cells; **(L)** Histogram showing the number of SW1783 cells migrated under the co-culture system. **p < 0.01, and ***p < 0.001.

To further confirm our speculation above, we induced human THP-1 monocytes into M2 macrophages under established conditions. RT-qPCR results also demonstrated that the induced M2 macrophages expressed higher levels of M2 markers CD86, CD206, and ARG1 (**Figures 9A, B**). In addition, we also found that M2 macrophages expressed more IFI30 compared to THP-1 cells (**Figure 9C**). To further demonstrate the function of M2 macrophages in the malignant progression of GBM, we constructed a co-culture system in which we were able to visualize a significant increase in the expression level of IFI30 after co-culture with GBM cells (**Figure 9D**). In addition, scratch and migration assays demonstrated that GBM cells co-cultured with M2 macrophages acquired a higher migration and invasion capacity (**Figures 9E–H**). We then interfered with the expression level of IFI30 in the current co-culture system and demonstrated that although co-culture with M2 macrophages increased the viability of GBM cells, this malignancy was greatly reduced when the expression level of IFI30 was decreased, which more or less confirms that IFI30 is capable of interacting with M2 macrophages and thus influencing the growth of GBM cells (**Figures 9I–L**). In conclusion, these results demonstrated that IFI30 is essential for the generation of TAM with M2 phenotype in GBM.

## 4 Discussion

In sharp contrast to conventional chemotherapy and radiotherapy, the latter are often beset by a host of adverse side effects. However, tumor immunotherapy has emerged as a paragon of innovation, kindling high expectations within the medical community. By virtue of its unique mechanism of action—which hinges on harnessing the body’s endogenous immune system to target and eliminate tumor cells—it has transformed into a powerful driving force, inspiring researchers to overcome the existing problem of the poor efficacy of immune checkpoint inhibitors (ICI) in certain patient groups (31). Recently, successive evidence confirmed that immunosuppression due to interactions between TME cells is a major obstacle to the clinical potential of immunotherapy, where the dynamic evolution of the M1-M2 phenotype of macrophages largely contributes to the immune signaling (32). On the one hand, while they can promote tumor immunity by transforming into the M1 subtype and releasing cytotoxically active cytokines, on the other hand, mainly the M2 subtype promotes tumor proliferation, angiogenesis, and distant metastasis by boosting the formation of a tumor immunosuppressive microenvironment (33). The blockage of M2-macrophage recruitment, depletion of M2 macrophages or repolarization to the M1 state constitute the prevailing therapeutic strategies, where finding effective therapeutic targets or key genes is a proven means (33, 34). Systematic pan-cancer analysis of IFI30 by combining transcriptomic as well as single-cell sequencing data in the current study might to a certain extent point the way to such a puzzle.

Indeed, as demonstrated in this study, IFI30 was significantly more expressed in the majority of all cancers than in normal controls, suggesting a critical player in the progression of cancer, as supported by the prognostic and diagnostic value of the KM survival curve and the ROC curve, respectively. In addition, we maximized the breadth of transcriptomic data to predict the potential biological functional pathways of IFI30 in different tumors. Although IFI30 was significantly associated with all of the included Hallmarks pathways, we found that pathways such as TNF, IFN and others rendered significant co-association in almost all of the tumors included in the study. The current consensus that inflammatory responses, TNFA signaling, and IFN signaling correlate with immunotherapy in cancer and patient response implies that the potential immunotherapeutic value of IFI30 could be explored through these pathways, which also substantiates our previous hypothesis (35–37). In addition, we directed our attention to the immunotherapeutic pathways in the GO-BP dataset, and similarly identified pathways that are co-responsive in pan-cancer, such as activation/regulation of immune pathways that affect T-cell function, among others. Unlike T cells, which have become the hot star cells due to chimeric antigen receptor (CAR) T-cell therapy, NK cells, as a specific immune effector cell capable of relatively simple and rapid immune response, have been demonstrated to be critical in immune activation against abnormal cells, which has also gained widespread attention in the field of cancer immunotherapy (38, 39). A bridge to optimize these therapeutic measures could be built in the future through IFI30.

The further assessment of the relevance of IFI30 to immune infiltration scores further sheds light on the potential immunotherapeutic value. All the three immune scores selected for this study presented a significant positive correlation with IFI30. The positive correlation was particularly prominent in cancers such as GBM, LGG, TGCT, SKCM, PAAD, DLBC, and BLCA, which suggests a possible place of interest in the immune microenvironment of these tumors. With this study we have tried to explore this possibility by correlation analysis of ICB-related genes, MHC-related genes, or immunotherapeutic influences, such as TMB and MSI. The immune checkpoints serve as accessory molecules that function importantly in promoting or inhibiting T cell activation, and the destruction of negative regulatory checkpoints with antibodies that restore the immunosuppressed state of TME and release antitumor responses has been clinically evidenced to be effective in a variety of forms of cancer (40–42). Similarly, vital immune checkpoint genes such as HAVR2, CD274, CTLA4, ITGB2, ICAM1, CCL5, CXCL10 all exhibited robust correlation with IFI30, and this immunotherapy might, in the future, establish a bond with IFI30, bringing new opportunities for cancer treatment. When further placing the contribution of IFI30 in the immunotherapeutic setting, evaluation is required for the effect of immunotherapy as well. TMB, MSI is thought to impact the efficacy of treatment with immune checkpoints (43). Our study confirmed a significant trend of correlation between IFI30 and TMB, MSI, and this correlation exhibited consistency with the prognostic analysis of IFI30, suggesting that in-depth consideration of immune markers is an equally critical factor influencing future immunotherapy decisions. We likewise evaluated the relationship between IFI30 and immune modulators, which was similarly significant, whereby MHC-related genes dominated. One mechanism by which tumor cells evade immune surveillance is through downregulation of the expression profile of major histocompatibility complex I (MHC-I), which has been described as a mechanism of intrinsic and acquired resistance to immunotherapy in cancer patients (44–46). To this we made the conjecture that the mechanism by which IFI30 affects immune properties may involve alterations in the MHC pathway. Another evidence for this is the existing studies confirming that TNFα strongly stimulates NFkB signaling and subsequent MHC expression, which echoes our GSEA analysis above, namely the confirmation that IFI30 is correlated with the TNFα pathway (47, 48). Based on the argument that tumor-infiltrating immune cells in TME take an influential role in cancer progression, we further analyzed the infiltration of IFI30 with a variety of immune cells. EPIC, TIMER, and MCP-counter each revealed a powerful correlation between IFI30 and immune infiltration from diverse perspectives, as reflected by the presence of T cells, B cells, and other cell subpopulations. The current study concluded that not only cancer cells are capable of immune escape with the aid of their own established complex microenvironment, but they are also competent to inhibit tumor progression and invasion owing to some immune cells in TIME (49, 50). Whether this paradoxical dilemma could be broken in the future by the intervention of IFI30 is unknown to us.

Our attention was drawn to the prognostic value of IFI30 in GBM, and for this reason, further analysis was performed. One noteworthy aspect is that the relationship between IFI30 and macrophages was also validated by several GBM single-cell datasets, for which we made the conjecture that IFI30 may be able to influence the survival status of M2 cells and thus exacerbate the malignant progression of GBM cells, as shown in subsequent cell communication analysis as well as laboratory work was confirmed. In fact, most TAMs fall into the role of supporting tumor cell growth and metastasis while losing their ability to fight tumor progression, whose help to establish an immune dysfunctional microenvironment by secreting many immunosuppressive cytokines (51). TAM-targeted therapy enhances the efficacy of immune checkpoint blockade therapies and complements the role of cancer checkpoint immunotherapy, linking the two therapies Together, the two therapies are expected to become an emerging tool for cancer immunotherapy (52, 53). In this process, the maximal exploitation of the contribution of IFI30 seems to offer a ray of hope for the current immunotherapy dilemma. In addition, the current study has only confirmed the effect of IFI30 on macrophages, but it is crucial to further dissect this property from upstream or downstream mechanisms in the future, and further linking the regulation of multiple signaling pathways and TAM repolarization strategies in combination with the exosomes, bacterial therapies, NPs and CAR-M therapies would make our research burst into renewed splendor (32, 54, 55).

## Data Availability

The original contributions presented in the study are included in the article/**Supplementary Material**. Further inquiries can be directed to the corresponding author.

## Supplementary material

Supplementary Figure 1 Exploration of the underlying immunotherapeutic potential of IFI30. **(A)** Relationship between IFI30 and StromalScore; **(B)** Relationship between IFI30 and ImmuneScore; **(C)** Relationship between IFI30 and EstimateScore; **(D)** Correlation between IFI30 expression and immune checkpoint-related genes; **(E)** Correlation analysis between IFI30 expression and TMB in diverse tumors; **(F)** The correlation analysis of IFI30 expression with MSI in different tumors.

Supplementary Figure 2 Significant correlation analysis of IFI30 with immune-related factors at the pan-cancer level.

Supplementary Figure 3 Details of Quality Control for Spatial Transcriptomics Data.
