# Supplementary figures and images for: Comprehensive single-cell pan-cancer atlas unveils IFI30+ macrophages as key modulators of intra-tumoral immune dynamics
Source: Front Immunol. 2025 Jan 24;16:1523854. doi: 10.3389/fimmu.2025.1523854 (PMC11802554; doi:10.3389/fimmu.2025.1523854)

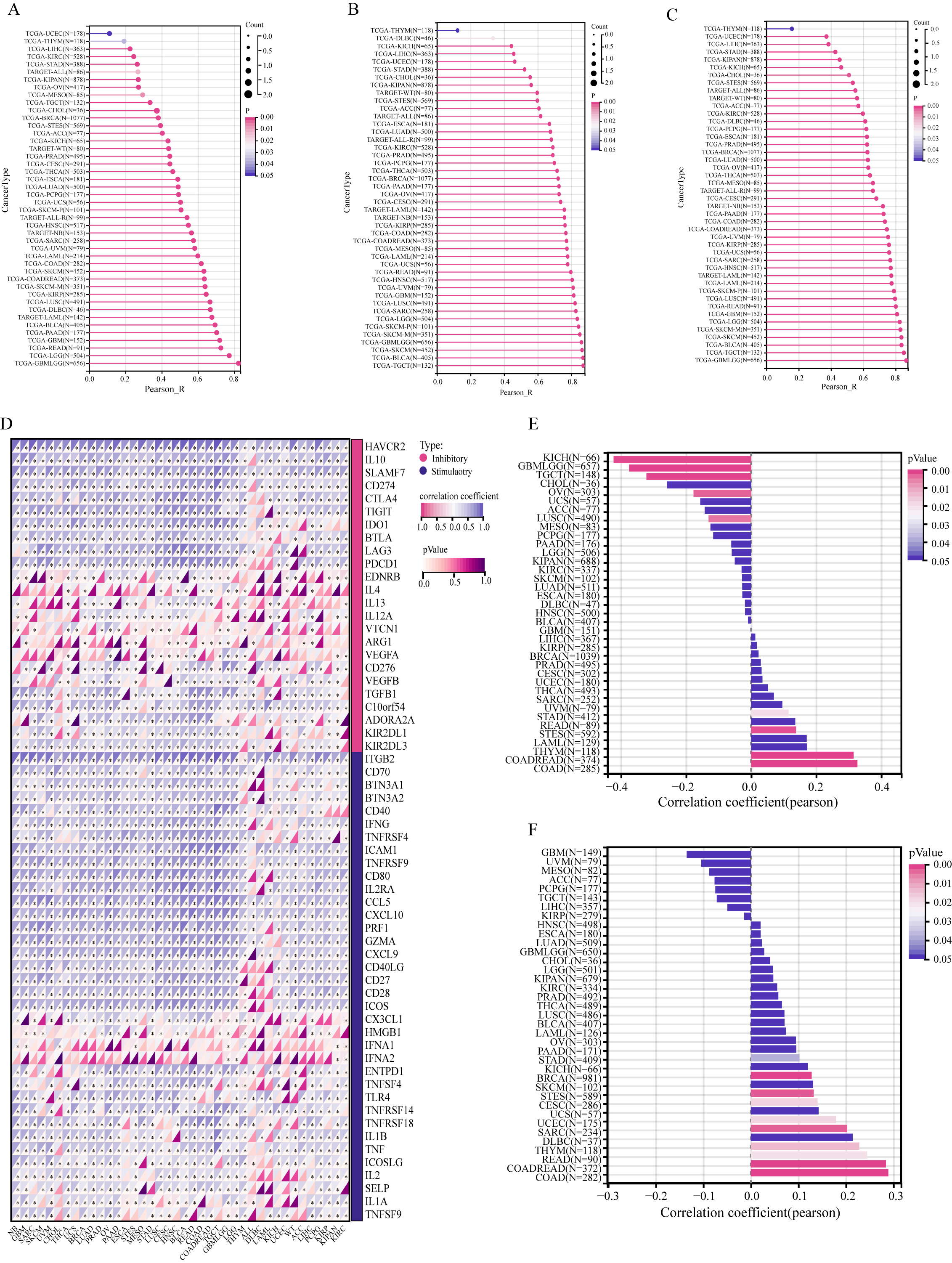

Supplement: Supplementary Figure 1 — Exploration of the underlying immunotherapeutic potential of IFI30. (A) Relationship between IFI30 and StromalScore; (B) Relationship between IFI30 and ImmuneScore; (C) Relationship between IFI30 and EstimateScore; (D) Correlation between IFI30 expression and immune checkpoint-related genes; (E) Correlation analysis between IFI30 expression and TMB in diverse tumors; (F) The correlation analysis of IFI30 expression with MSI in different tumors. [file Image1.tif]

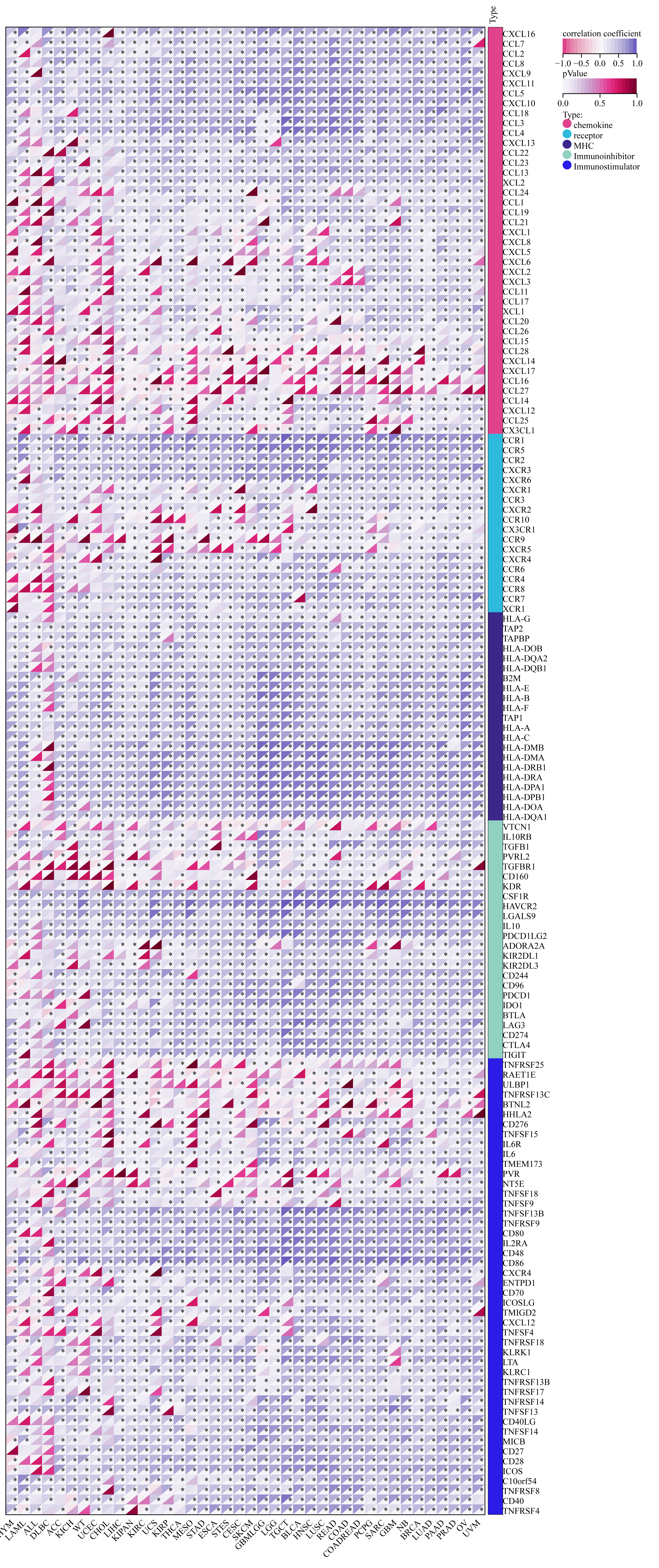

Supplement: Supplementary Figure 2 — Significant correlation analysis of IFI30 with immune-related factors at the pan-cancer level. [file Image2.tif]

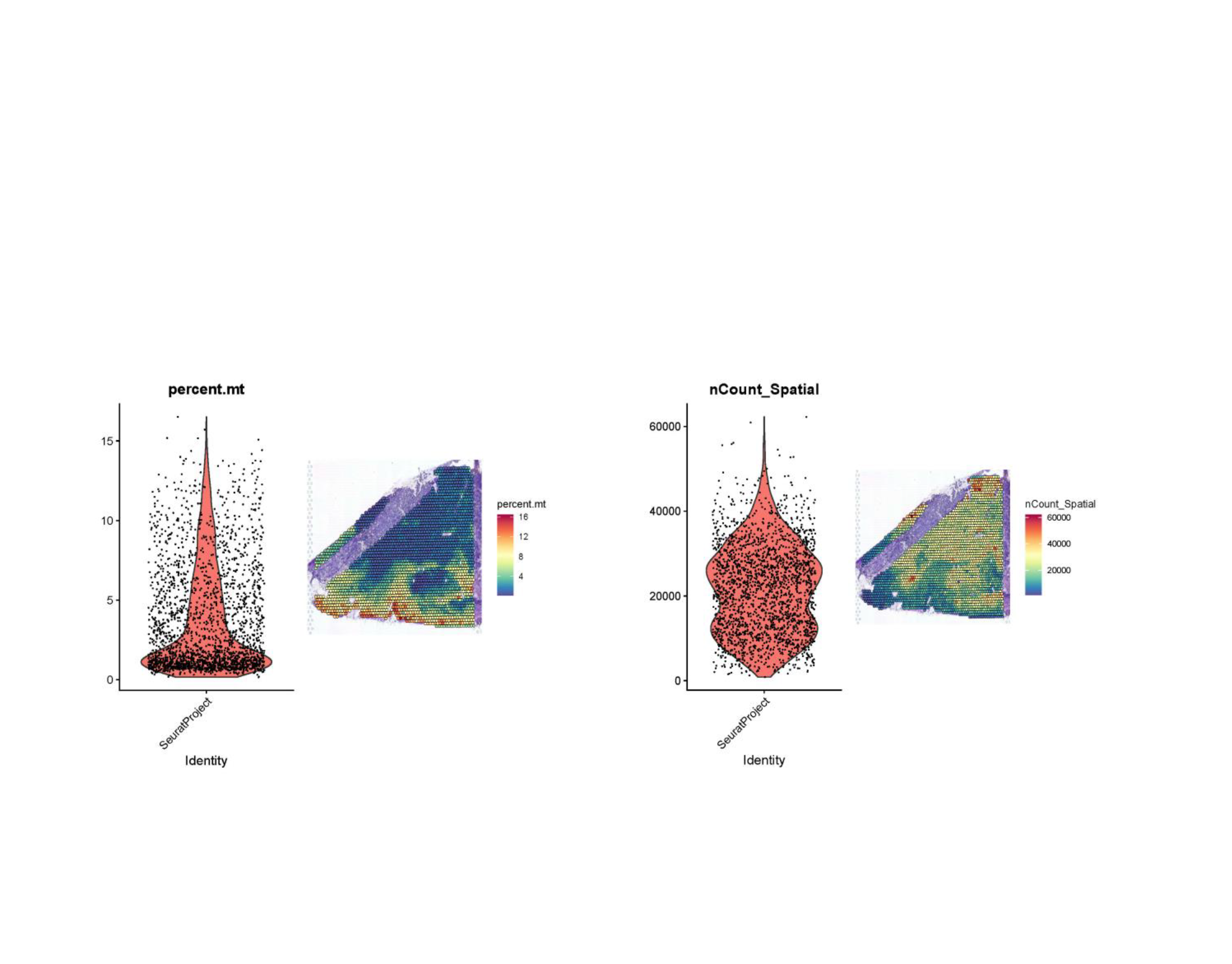

Supplement: Supplementary Figure 3 — Details of Quality Control for Spatial Transcriptomics Data. [file Image3.tif]
