# Supplementary material for: Comprehensive single-cell pan-cancer atlas unveils IFI30+ macrophages as key modulators of intra-tumoral immune dynamics
Source: Front Immunol. 2025 Jan 24;16:1523854. doi: 10.3389/fimmu.2025.1523854 (PMC11802554; doi:10.3389/fimmu.2025.1523854)
Supplement: Supplementary file 4 [file DataSheet1.pdf]

Summary of the pan-cancer dataset

Cancer_type	Full name of cancer_type	Sample size	Age		Gender		Tumor_stage				
			<60	⩾60	Female	Male	I	Ⅱ	III	IV	Unknown
ACC	Adrenocortical cancer	77	57	20	46	31	9	36	15	15	2
BLCA	Bladder urothelial carcinoma	426	91	335	115	311	2	134	147	141	2
BRCA	Breast invasive carcinoma	1211	652	559	1198	13	202	687	275	22	25
CESC	Cervical & endocervical cancer	309	243	66	309	0	0	0	0	0	309
CHOL	Cholangiocarcinoma	45	13	32	23	22	26	10	1	8	0
COAD	Colon adenocarcinoma	329	101	228	153	176	48	133	90	47	11
DLBC	Diffuse large B-cell lymphoma	47	26	21	25	22	0	0	0	0	47
ESCA	Esophageal carcinoma	195	87	108	31	164	24	79	57	9	26
GBM	Glioblastoma	165	78	87	59	106	0	0	0	0	165
HNSC	Head & neck squamous cell carcinoma	564	248	316	151	413	29	86	88	285	76
KICH	Kidney chromophobe	91	64	27	39	52	31	33	17	10	0
KIRC	Kidney clear cell carcinoma	603	272	331	206	397	292	68	139	101	3
KIRP	Kidney papillary cell carcinoma	321	133	188	86	235	185	23	64	19	30
LAML	Acute myeloid leukemia	173	90	83	80	93	0	0	0	0	173
LGG	Lower grade glioma	522	452	70	233	289	0	0	0	0	522
LIHC	Liver hepatocellular carcinoma	421	185	236	142	279	188	98	97	6	32
LUAD	Lung adenocarcinoma	574	154	420	310	264	306	135	96	28	9
LUSC	Lung squamous cell carcinoma	548	99	449	143	405	269	178	89	8	4
MESO	Mesothelioma	87	27	60	16	71	10	16	45	16	0
OV	Ovarian serous cystadenocarcinoma	427	225	202	427	0	0	0	0	0	427
PAAD	Pancreatic adenocarcinoma	183	58	125	82	101	21	151	3	5	3
PCPG	Pheochromocytoma	185	143	42	102	83	0	0	0	0	185
PRAD	Prostate adenocarcinoma	548	223	325	0	548	0	0	0	0	548
READ	Rectum adenocarcinoma	102	43	59	49	53	16	26	35	15	10
SARC	Sarcoma	264	119	145	145	119	0	0	0	0	264
SKCM	Skin cutaneous melanoma	470	244	226	180	290	77	140	169	24	60
STAD	Stomach adenocarcinoma	450	129	321	159	291	64	140	176	44	26
TGCT	Testicular germ cell tumor	137	135	2	0	137	54	12	14	0	57
THCA	Thyroid carcinoma	571	435	136	414	157	324	59	125	61	2
THYM	Thymoma	121	60	61	59	62	0	0	0	0	121
UCEC	Uterine corpus endometrioid carcinoma	194	54	140	194	0	0	0	0	0	194
UCS	Uterine Carcinosarcoma	57	6	51	57	0	0	0	0	0	57
UVM	Uveal melanoma	79	36	43	35	44	0	39	35	4	1
